# Understanding changes in echocardiographic parameters at different ages following fetal growth restriction: a systematic review and meta-analysis

**DOI:** 10.1152/ajpheart.00052.2024

**Published:** 2024-04-26

**Authors:** Mette van de Meent, Kirsten T. Nijholt, Shary C. A. Joemmanbaks, Judith Kooiman, Henk S. Schipper, Kimberley E. Wever, A. Titia Lely, Fieke Terstappen

**Affiliations:** ^1^Division Women and Baby, Department of Obstetrics, Wilhelmina Children’s Hospital, Utrecht University, Utrecht, The Netherlands; ^2^Department of Pediatric Cardiology, Sophia Children’s Hospital, Erasmus Medical Center Rotterdam, Rotterdam, The Netherlands; ^3^Department of Anesthesiology, Pain and Palliative Medicine, Radboud University Medical Center, Nijmegen, The Netherlands; ^4^Division Women and Baby, Department of Neonatology, Wilhelmina Children’s Hospital, Utrecht University, Utrecht, The Netherlands

**Keywords:** echocardiography, fetal growth restriction, meta-analysis, small for gestational age

## Abstract

Fetal growth restriction (FGR) increases cardiovascular risk by cardiac remodeling and programming. This systematic review and meta-analysis across species examines the use of echocardiography in FGR offspring at different ages. PubMed and Embase.com were searched for animal and human studies reporting on echocardiographic parameters in placental insufficiency-induced FGR offspring. We included six animal and 49 human studies. Although unable to perform a meta-analysis of animal studies because of insufficient number of studies per individual outcome, all studies showed left ventricular dysfunction. Our meta-analyses of human studies revealed a reduced left ventricular mass, interventricular septum thickness, mitral annular peak velocity, and mitral lateral early diastolic velocity at neonatal age. No echocardiographic differences during childhood were observed, although the small age range and number of studies limited these analyses. Only two studies at adult age were performed. Meta-regression on other influential factors was not possible due to underreporting. The few studies on myocardial strain analysis showed small changes in global longitudinal strain in FGR offspring. The quality of the human studies was considered low and the risk of bias in animal studies was mostly unclear. Echocardiography may offer a noninvasive tool to detect early signs of cardiovascular predisposition following FGR. Clinical implementation yet faces multiple challenges including identification of the most optimal timing and the exact relation to long-term cardiovascular function in which echocardiography alone might be limited to reflect a child’s vascular status. Future research should focus on myocardial strain analysis and the combination of other (non)imaging techniques for an improved risk estimation.

## INTRODUCTION

Cardiovascular disease remains the leading cause of mortality in adults worldwide (1). Although the cardiovascular burden clearly manifests during adulthood, the predisposition of cardiovascular disease starts already during fetal development (2, 3). Cardiac remodeling and programming in utero can be negatively influenced by exposure to adverse early life events (2, 4). The exposure to other unhealthy behavioral (e.g., smoking) or biological factors (e.g., untreated hypertension) throughout the life span add to the cardiovascular risk profile, whereby subclinical changes might progress into cardiovascular disease in adulthood (5).

Fetal growth restriction (FGR) is one of these adverse early life events that has been associated with increased susceptibility to cardiovascular disease by fetal programming (2). This common complication of pregnancies arises from placental insufficiency in which a reduced supply of nutrients and oxygen hampers the fetus from reaching its intrinsic growth potential (6, 7). Chronic hypoxia initiates endothelial dysfunction and fetal hemodynamic redistribution. In addition, the increased placental resistance results in persistent volume/pressure overload in the fetal heart (8). Cardiac remodeling occurs with the purpose of maintaining output in which the phenotype depends on the severity (8). Increasing evidence shows cardiac remodeling on both macroscopic and microscopic levels in growth-restricted fetuses that persists after birth (4, 8–10). This fetal cardiac remodeling and adverse programming may underlie the observed increased cardiovascular risk in the FGR population.

Early identification of high-risk patients provides the opportunity to prevent or minimize the cardiovascular health burden from the start of life. Acknowledgment of this window of opportunity led to a shift in approach toward prevention as a key strategy in cardiovascular health management over the life span (11). Longitudinal follow-up of cardiac remodeling after birth provides insight into the progression from subclinical to clinical signs of cardiovascular disease secondary to fetal growth restriction. This gradual process is currently insufficiently understood and depends on several factors including sex and severity of FGR. In addition, the dynamic aspects make the moment of measurement essential when researching this topic. For instance, hypertrophic remodeling has been observed more frequently in early-onset FGR while late-onset FGR mostly shows less severe cardiac remodeling (8). A better understanding of effect modifiers allows identification of the subgroups most at risk and simultaneously who will benefit most from perinatal interventions or early screening programs. However, longitudinal assessment of cardiovascular health in the pediatric population remains a relatively uncharted territory.

Echocardiography is the golden standard for evaluating cardiac development and function at different ages in a noninvasive way. Novel ultrasound techniques such as speckle-tracking have recently augmented the potential of conventional ultrasound to detect subclinical changes in structure and function as an early biomarker for cardiovascular predisposition (12–14). Both clinical and animal studies have shown differences in cardiac development following FGR (8). Although cardiac ultrasound presents a unique opportunity to improve cardiovascular outcomes in growing numbers of children and adolescents at risk, a current overview of (sub)clinical progression over the life span and effect modifiers is lacking.

This systematic review and meta-analysis across species examine the use of cardiac ultrasound in fetal growth-restricted offspring as a consequence of placental insufficiency. We aim to understand echocardiographic changes during different ages following FGR. An improved understanding of the role of echocardiography could eventually contribute to the ongoing development of cardiovascular screening programs in FGR offspring.

## METHODS

### Study Protocol

This systematic review was conducted according to a protocol preregistered on PROSPERO (CRD42022330269) and reported following the Preferred Reporting Items for Systematic Reviews and Meta-Analyses guidelines (Supplemental Table S1; all Supplemental Material is available at https://doi.org/10.6084/m9.figshare.25479805.v2) (15).

### Literature Search

We searched PubMed and Embase.com from inception up to March 4, 2024, to identify animal and human studies reporting on cardiac ultrasound measurements in fetal growth-restricted offspring. The search string is provided in the supplemental data (Supplemental Table S2). No publication dates or language restrictions were applied.

### Inclusion and Exclusion Criteria

Two independent researchers (MvdM and SJ) screened articles for inclusion using predefined inclusion and exclusion criteria using Rayyan (https://rayyan.ai/cite). Discrepancies were resolved by discussion and in case of no consensus by a third investigator (FT).

We included echocardiographic studies in fetal growth-restricted offspring after exposure to placental insufficiency or chronic hypoxia in singleton pregnant mammals. FGR in human studies was defined as an estimated fetal weight (EFW) or abdominal circumference below the 10th percentile or deviating growth of two time points with a minimal of at least 2 wk. Of note, this definition of FGR was formulated by a Delphi procedure in 2016, and we did not want to exclude human studies published before 2016. We therefore broadened the definition of the human study population to include small for gestational age (SGA), which is defined as birth weight below the 10th percentile for gestational age. Studies including low birth weight without correction for gestational age were excluded as this population is either prematurity or a combination of prematurity and SGA wherefore the solitary effect of FGR could not be investigated. Induction methods of FGR in animals included surgical ligation of uterine vessels, chronic hypoxia exposure, and genetic predisposition [i.e., Dahl salt-sensitive (SS) rats (16) or spontaneous hypertension and heart failure (SHHF) (17) rats (18)].

Studies on FGR caused by diet restriction or genetic modeling were excluded as the induction of these models lacks the exposure of chronic hypoxia and thereby the pathophysiological sequala of hemodynamic redistribution and cardiac remodeling (6, 7). Other exclusion criteria were lack of a control group and lack of original data (e.g., narrative review). For human studies, we included observational cohort studies, randomized controlled trials (RCT), or case-control trials.

### Data Extraction

Two researchers (MvdM and KN) independently extracted data. Discrepancies were resolved by discussion in case of no consensus by a third investigator (FT). All echocardiographic data and information on study design were extracted including birth weight, sex of offspring, age, and conditions during measurements. In addition for animal studies, we extracted data on species, strain, animal model, and litter size, and for human studies also ethnicity, BMI, and coexistence of other hypertensive disorders of pregnancy. Per primary outcome, we noted for all echocardiographic measurements including speckle-tracking measurements: means (SD) and number of subjects per group. Secondary outcomes included other cardiovascular parameters, for example, blood pressure and heart rate. The corresponding authors were contacted once by email in case of missing data.

### Assessment of Risk of Bias and Study Quality

The risk of bias was assessed by two independent researchers (MvM and KN). Discrepancies were resolved by discussion and by a third investigator (FT or KW). For animal studies, we used the risk of bias tools from Systematic Review Center for Laboratory Animal Experimentation (SYRCLE) (19) and the Newcastle-Ottawa Scale for human cohort studies (20). Adjustments to the tools are described in the supplement (Supplemental Method). Studies were not excluded based on poor study quality.

### Meta-Analysis

Meta-analyses across species were performed for each echocardiographic outcome reported in more than five studies in a similar age group (these age categories are defined below). The pooled effect size estimates were presented as standardized mean difference (SMD) with their 95% confidence intervals (95%CI). First, we explored the potential difference in the pooled effect size caused by species to decide whether pooling of data was justified. We applied a random effects model because of potential heterogeneity in animal studies with various species and strains, FGR induction methods, and different units of measurement reported. We used nesting if multiple cohorts from one study were included. In case of multiple measurements at different ages as part of a follow-up study, we used only the latest measurement within the same age category for the meta-analysis.

Meta-regression analysis allows exploring the impact of certain study characteristics on the observed effect size, e.g., determining sources of heterogeneity. We only performed meta-regression when at least two of the subgroups contained at least 10 studies per age category. The following groups were predefined; species (human vs. rat vs. mice), severity of FGR (in percentiles [p]: <p3 vs. p3–p10), method of FGR induction (surgical vs. hypoxic chamber), sex (male vs. female), and age at cardiac ultrasound assessment (neonate vs. child vs. young adult). Definition of these age categories included neonates from birth until 28 days after birth, children from 29 days after birth until 17 yr old, and adults as 18 yr and older.

Statistical analyses were performed using R software (v. 4.2.3, Auckland, New Zealand, Metafor package). A two-sided *P* value < 0.05 was considered significant. For outcomes reported in at least 20 studies, we produced funnel plots and Egger’s regression test to assess publication bias. Heterogeneity (I^2^) among studies of 25% was considered low, 25–50% as moderate, 50–75% as high, and 75–100% as very high, in accordance with the Cochrane Handbook of Systematic Reviews of Interventions (21).

## RESULTS

### Study Selection

Our search retrieved 7,926 records (Supplemental Fig. S1). Screening resulted in a total of 55 included studies in this systematic review and meta-analysis. A complete list of exclusion criteria is reported in the supplemental data (Supplemental Table S3). Exclusion of studies was mostly based on the lack of the correct study groups (both FGR or control group), followed by missing an outcome of interest. Because of the very limited amount of animal study data available in the various age groups, the suitability of pooling animal and human data could not be assessed. Consequently, to avoid inappropriate pooling across species, only human data were included in our meta-analysis. Therefore, and according to our predefined protocol, we analyzed and presented the results separately for humans (Table 1) and animals (Table 2).

**Table 1.** Overview of the main study characteristics and results of included human studies

Author (Year)	FGR Definition	FGR, *n*	Control, *n*	Age	Significant Differences	Nonsignificant Results
Aburawi (2011) (22)	BW > −2 SD	17	15	1 wk	Cardiac morphology: LVMI increased in FGR. Coronary flow: Basal and peak flow velocity increased in FGR.	Cardiac morphology: LV end-diastolic dimension, LVM. Systolic function: Cardiac output, mitral peak early wave, fractional shortening.
Akazawa (2016) (23)	BW < p10	38	30	1 wk	Cardiac morphology: LV diastolic dimensions decreased in SGA. Systolic function: stroke volume, left cardiac output, septal s′ peak velocity, mitral lateral s′ increased in SGA, global longitudinal strain decreased in SGA. Diastolic function: mitral annular early diastolic velocity decreased in SGA, isovolumetric relaxation time increased in severe SGA.	Cardiac morphology: LV posterior wall, RWT, LVMI. Systolic function: fractional shortening, left cardiac output index, tricuspid lateral s′, global circumferential strain. Diastolic function: mitral E wave, mitral A wave, mitral E/A ratio, tricuspid E wave, tricuspid A wave, tricuspid E/A ratio, mitral E deceleration time, tricuspid e′, E/e′ (LV lateral), E/e′ (septal), E/e′ (RV), MPI.
Akazawa (2018) (24)	BW < p10	31	27	1 wk	Cardiac morphology: LV diastolic dimension decreased in SGA, LV mass index increased in SGA. Systolic function: fractional shortening increased in SGA, stroke volume, left cardiac output, mitral lateral annular systolic (S′) decreased in SGA. Diastolic function: left isovolumic relaxation time increased in SGA, mitral early diastolic lateral (E′) tissue Doppler velocity decreased in SGA.	Cardiac morphology: LVM. Systolic function: left cardiac output index, Septal S′ peak velocity. Diastolic function: mitral E wave, mitral A wave, mitral E/A ratio, mitral E deceleration time, mitral septal E′ tissue Doppler velocity.
Altin (2012) (25)	BW > −2 SD	30	30	72 h postpartum	Cardiac morphology: IVSd, LVPWTd, LVEDd, LVESd, and LVOTd decreased in SGA. LVEDdI increased in SGA. Systolic function: LV stroke volume, LV cardiac output, LV myocardial systolic peak velocity, RV myocardial systolic peak velocity, IVS myocardial peak velocity decreased in SGA. LV cardiac index increased in SGA. Diastolic function: LV E/A increased. LV Em/Am, RV Am decreased.	Systolic function: EF, fractional shortening, LV stroke index. Diastolic function: LV E, LV A, RV E, RV A, RV E/A, LV Em, LV Am, LV E/Em, RV Em, RV Em/Am, RV E/Em, IVS Em, IVS Am, IVS Em/Am.
				3 mo	Cardiac morphology: LVPWTd, LVEDd, LVESd, LVOTd decreased in SGA. LVEDdI increased in SGA. Systolic function: LV stroke volume, LV cardiac output decreased in SGA. LV cardiac index increased in SGA.	Cardiac morphology: IVSd. Systolic function: LVEF, fractional shortening, LV stroke index, LV myocardial systolic peak velocity, RV myocardial systolic peak velocity, IVS myocardial peak velocity. Diastolic function: LV E, LV A, LV E/A, RV E, RV A, RV E/A, LV Em, LV Am, LV Em/Am, LV E/Em, RV Em, RV Am, RV Em/Am, RV E/Em, IVS Em, IVS Am, IVS Em/Am.
				6 mo	Cardiac morphology: LVEDdI and increased in SGA. Systolic function: LV stroke volume decreased in SGA. LV cardiac index increased in SGA.	Cardiac morphology: IVSd, LVPWTd, LVEDd, LVOTd. Systolic function: EF, fractional shortening, LV stroke index, LV cardiac output, LV myocardial systolic peak velocity, RV myocardial systolic peak velocity, IVS myocardial peak velocity. Diastolic function: LV E, LV A, LV E/A, RV E, RV A, RV E/A, LV Em, LV Am, LV Em/Am, LV E/Em, RV Em, RV Am, RV Em/Am, RV E/Em, IVS Em, IVS Am, IVS Em/Am.
Änhagen (2022) (12)	BW > −2.5 SD or 22% lower fetal weight than predicted for GA and/or 10% or greater drop in fetal growth velocity based on the weight that would be predicted from the most recent ultrasound examination	19	35	12–72-h postpartum	Left ventricle: LVEDd, IVSd, LVPWTd, LV mass, LV mass index, VL septum systole, VL septum diastole, sphericity index increased. Right ventricle: DL septum, VL septum systole, DL free wall, VL free wall systole, VL free wall diastole increased.	Left ventricle: RWT, DL septum, DL lateral wall, VL lateral wall systole, VL lateral wall diastole, average four segment SL, average SL septal wall, average SL lateral wall. Right ventricle: VL septum diastole.
				3–4 mo	Left ventricle*:* LV mass index increased. DL septum, VL septum diastole, VL septum systole, DL lateral wall, VL lateral wall systole, VL lateral wall diastole, average four segment SL, average SL septal wall, average SL lateral wall, sphericity index decreased. Right ventricle: DL septum, VL septum diastole decreased.	Left ventricle: LVEDd, IVSd, LVPWd, LVM, RWT. Right ventricle: VL septum systole, DL free wall, VL free wall systole, VL free wall diastole.
Arnott (2015) (26)	BW < p10	157	627	34–49 yr	Cardiac morphology (indexed for BSA): LVEDD, LVESD, LV basal diameter, RV base-to-apex increased. Systolic function (adjusted for age, sex, blood pressure, physical activity levels, and socioeconomic status): LV stroke volume decreased.	Cardiac morphology (indexed for BSA): Left atrial area, left atrial volume, right atrial area, right atrial volume, LV end-systolic volume, LV end-diastolic volume, LVM, RV basal diameter, RV end-systolic volume, RV end-diastolic volume. Systolic function (adjusted for age, sex, blood pressure, physical activity levels, and socioeconomic status): LV S′ lateral, LV stroke volume, EF, LV cardiac output, LV fractional area change, RV fractional area change. Diastolic function (adjusted for age, sex, blood pressure, physical activity levels, and socioeconomic status): LV E wave, LV A wave, LV E′ medial, LV E′ lateral, LV E/E′, LV deceleration time.
Bjarnegård (2013) (27)	BW > −2.5 SD	19	18	23 yr	Cardiac morphology: LVEDd decreased in FGR.	Cardiac morphology: Left atrium, IVSd, LVPWd, LVMI.
Castagno (2019) (28)	BW < p10	20	20	2 yr	Cardiac morphology: LVEDd, LVESd, LV area, LV volume decreased in SGA. LVPW, RWT increased in SGA. Systolic function: LV stroke volume, TAPSE decreased in SGA. Diastolic function: Mitral lateral E′ velocity decreased in SGA.	Cardiac morphology: IVSd, LVM, LVMI. Systolic function: Fractional shortening, LVEF, LV output. Diastolic function: Mitral E peak, mitral A peak, E/A ratio, E/E′ ratio.
Cinar (2013) (29)	BW < p10, ponderal index > 2.25 (symmetric)	39	50	48–72-h postpartum	Cardiac morphology: LVEDd, LVPWd, LVWPDs, RWT, LVM, LVMI decreased in FGR.	Cardiac morphology: IVSd, IVSs, LVESd, LVPWs. Systolic function: LVEF, fractional shortening.
	BW < p10, ponderal index < 2.25 (asymmetric)	62	50	48–72-h postpartum	Cardiac morphology: LVEDd, LVESd, LVPWd, LVPWs, RWT, LVM, LVMI decreased in FGR.	Cardiac morphology: IVSd, IVSs. Systolic function: LVEF, fractional shortening.
Cohen (2017) (30)	EFW < p10 and/or fetal growth plus abnormal Doppler	24	23 preterm 19 term	24 h	Cardiac morphology: 3-D sphericity index increased in FGR.	Cardiac morphology: 2-D sphericity index, LVPWd, RWT. Diastolic function: Lateral E/e′ ratio, medial E/e′ ratio.
				1 mo	Cardiac morphology: 2-D sphericity index decreased in FGR. 3-D sphericity index increased in FGR. Diastolic function: Medial and lateral E/e′ ratio decreased in FGR.	Cardiac morphology: LVPWd, RWT.
				6 mo	No significant differences.	Cardiac morphology: 2-D sphericity index, 3-D sphericity index, LVPWd, RWT. Diastolic function: Lateral E/e′ ratio, medial E/e′ ratio.
Crispi (2010) (31)	BW < p10 with normal PI UA	40	120	5 yr	Cardiac morphology: Base-to-apex length LV decreased in FGR. Basal diameter LV and RV, sphericity index LV and RV increased in FGR. Systolic function: Mitral septal S′, E/E′ (lateral), E/E′ (septal) decreased in FGR. Left cardiac output increased in FGR. Diastolic function: Mitral E deceleration time, tricuspid E deceleration time increased in FGR.	Cardiac morphology: IVSd, LVPWd, RWT. Systolic function: Left stroke volume, LVEF, mitral lateral S′, tricuspid S′. Diastolic function: Mitral E wave, mitral A wave, mitral E/A ratio, tricuspid E wave, tricuspid A wave, tricuspid E/A ratio, left isovolumic relaxation time, tricuspid E deceleration time, mitral lateral E′, mitral septal E′, tricuspid E′, E/E′ (lateral), E/E′ (septal).
	BW < p10 with abnormal (PI > 2SDs)	40	120	5 yr	Cardiac morphology: Basal diameter LV, sphericity index LV, and RV increased in FGR. Systolic function: Left stroke volume, mitral lateral S′, mitral septal S′, tricuspid S′ decreased in FGR. Diastolic function: Mitral E deceleration time, tricuspid E deceleration time E/E′ (lateral), E/E′ (septal) increased in FGR. Mitral lateral E′, mitral septal E′, tricuspid E′ decreased in FGR.	Cardiac morphology: IVSd, LVPWd, RWT. Systolic function: LVEF. Diastolic function: Mitral E wave, mitral A wave, mitral E/A ratio, tricuspid A wave, tricuspid E/A ratio, left isovolumic relaxation time.
Crispi (2012) (32)	BW p3–p10 with normal CPR	25	100	5 yr	Cardiac morphology: left sphericity index decreased in FGR. Systolic function: normalized left stroke volume, MAPSE decreased in FGR. Normalized left cardiac output, left ventricular thickening increased in FGR. Diastolic function: Mitral isovolumic relaxation time′.	Cardiac morphology: Right sphericity index, RWT. Systolic function: LVEF, mitral S′, TAPSE, tricuspid S′. Diastolic function: Mitral E deceleration time, mitral E/A ratio, mitral E′, mitral A′, mitral E′/A′, left isovolumic relaxation time, E/E′, tricuspid E deceleration time, tricuspid E/A ratio, tricuspid E′, tricuspid A′, tricuspid E′/A′, tricuspid isovolumic relaxation time′.
	BW < p3 or abnormal CPR	25	100	4 yr	Cardiac morphology: left and right sphericity index decreased in FGR.Systolic function: normalized left stroke volume, tricuspid S′, MAPSE decreased in FGR. Normalized left cardiac output, left ventricular thickening increased in FGR. Diastolic function: mitral E deceleration time, mitral E′/A′ increased in FGR. Mitral A′ decreased in FGR.	Cardiac morphology: RWT. Systolic function: LVEF. Diastolic function: Mitral E/A ratio, mitral E′, mitral E′/A′, left isovolumic relaxation time, mitral isovolumic relaxation time′, E/E′, tricuspid E/A ratio, tricuspid E′, tricuspid A′, tricuspid E′/A′, tricuspid isovolumic relaxation time′.
Cruz–Lemini (2015) (10)	EFW and BW < p10	80	80	6.5 mo	Cardiac morphology: Left atrial area IVS increased in FGR. Left sphericity index decreased in FGR. Systolic function: right cardiac output, TAPSE, MAPSE, Tricuspid S′ decreased in FGR. Diastolic function: Left isovolumetric relaxation time, mitral E deceleration time, mitral A duration, tricuspid E deceleration time, tricuspid A duration increased in FGR. Mitral lateral E′, mitral lateral A′, tricuspid E/A ratio, tricuspid E′, tricuspid A′ decreased in FGR.	Systolic function: LVEF, left stroke volume, right stroke volume, left cardiac output. Diastolic function: Mitral E/A ratio.
Czernik (2013) (33)	BW < p3	19	21	2 days	Cardiac morphology: LVPWd, LVEDd, LVM, mitral valve increased in FGR. Systolic function: fractional shortening decreased in FGR.	Cardiac morphology: IVSd. Systolic function: left ventricular output.
				28 days	Cardiac morphology: Mitral valve increased in FGR.	Cardiac morphology: IVSd, LVPWd, LVM.Systolic function: fractional shortening, left ventricular output.
Elmakaty (2023) (34)	BW < p10	114	414	2–3 days	Cardiac morphology: IVSd, IVSs, LVIDd, LVIDs, LVPWd, LVPWs, LVM, LVMI decreased. Systolic function: LV fractional shortening decreased.	No nonsignificant differences.
Faienza (2016) (35)	BW and/or length < p3	27	25	10 yr	Systolic function: TAPSE decreased in FGR. LVEF, LV and RV Tei index increased in FGR. Diastolic function: E/e′ ratio decreased in FGR. Left and right E/A ratio increased in FGR.	No nonsignificant differences.
Fouzas (2014) (36)	BW < p10 who suffered IUGR documented by fetal ultrasound biometry and umbilical artery Doppler	30	30	2 days	Cardiac morphology: IVSd, LVIDd, LVPWTd, LA, RWT decreased in FGR. IVSd/LVPWd ratio increased in FGR. Systolic function: LV stroke volume, LV cardiac output, LV and RV IVCT′ increased in FGR. Mitral lateral S′, tricuspid lateral S′ decreased in FGR. Diastolic function: Mitral E′, mitral E′/A′ ratio, tricuspid E′, tricuspid A′, tricuspid E′/A′ ratio decreased in FGR. LV IVRT, RV IVRT, LV E/E′ ratio, RV E/E′ ratio increased in FGR.	Systolic function: LV fractional shortening, LVEF. Diastolic function: Mitral E wave, mitral A wave, mitral E/A ratio, tricuspid E wave, tricuspid A wave, tricuspid E/A ratio, mitral A′, tricuspid A′, tricuspid E′/A′.
				5 days	Cardiac morphology: IVSd, LVPWTd, LA, RWT decreased in FGR. IVSd/LVPWTd ratio increased in FGR. Systolic function: LV stroke volume, mitral lateral S′, tricuspid lateral S′ decreased in FGR. LV IVCT′ increased in FGR. Diastolic function: Mitral E′, mitral E′/A′ ratio, tricuspid E′ decreased in FGR. RV IVRT′, LV E/E′ ratio, RV E/E′ ratio increased in FGR.	Cardiac morphology: LVIDd. Systolic function: LV fractional shortening, LVEF, LV cardiac output index, RV isovolumic contraction time′. Diastolic function: Mitral E wave, mitral A wave, mitral E/A ratio, tricuspid E wave, tricuspid A wave, tricuspid E/A ratio, mitral A′, tricuspid A′, tricuspid E′/A′.
Fouzas (2014) (37)	BW < p10 who suffered IUGR documented by fetal ultrasound biometry and umbilical artery Doppler	25	25	2 days	Cardiac morphology: IVSd, LVPWd, RWT decreased in FGR. IVSd/LVPWd increased in FGR. Systolic function: LV ICT and RV ICT increased in FGR. Diastolic function*:* Mitral E′, tricuspid E′ decreased in FGR. Mitral E/E′, tricuspid E/E′, LV IRT′, RV IRT′ increased in FGR.	Cardiac morphology: LVIDd.
				5 days	Cardiac morphology: LVPWd, RWT decreased in FGR. IVSd/LVPWd increased in FGR. Systolic function: LV ICT′, RV ICT′ increased in FGR. Diastolic function: Mitral E′, tricuspid E′ decreased in FGR. Mitral E/E′ tricuspid E/E′, LV IRT′, RV IRT′ increased in FGR.	Cardiac morphology: LVIDd.
Garofoli (2012) (38)		43	35	3, 30 days	No absolute values provided.	
Guajardo (1994) (39)	BW < p10	15	15	1 day	Systolic function: cardiac output increased in FGR.	No nonsignificant differences.
				5 days	No significant differences.	Systolic function: cardiac output.
Gürses (2013) (40)	Ponderal index < 10%	20	20	5 days	Cardiac morphology: LVPWd, LVEDd, IVSd, RVEDd, LA, LVM decreased in FGR.	Systolic function: LVEF, fractional shortening.
				1 mo	Cardiac morphology: LVEDd, RVEDd, LA, LVM decreased in FGR.	Cardiac morphology: LVPWd, IVSd. Systolic function: LVEF, fractional shortening.
				3 mo	Cardiac morphology*:* LVM decreased in FGR.	Cardiac morphology: LVPWd, LVEDd, IVSd, RVEDd, LA. Systolic function: LVEF, fractional shortening.
Harada (1998) (41)	BW < 2SD	13	29	4–6 days	Diastolic function: Peak E wave, peak A wave, peak E/A wave, velocity time integral of E wave, first third filling fraction, peak filling rate normalized to stroke volume, deceleration time decreased in FGR.	Diastolic function: Velocity time integral of A wave, velocity time integral of E/A.
Ishii (2013) (42)	BW < p10 with a head circumference within +2SD	30	57	3–6 h	Systolic function: LVEF, LV cardiac output decreased in FGR.	Diastolic function: E/e′.
				12 h	No significant differences.	Systolic function: LVEF, LV cardiac output. Diastolic function: E/e′.
				24 h	Systolic function: LVEF, LV cardiac output decreased in FGR.	Diastolic function: E/e′.
				48 h	Systolic function: LVEF, LV cardiac output decreased in FGR.	Diastolic function: E/e′.
				72 h	Systolic function: LVEF, LV cardiac output decreased in FGR.	Diastolic function: E/e′.
Ivanov (2019) (43)	BW p3–p10, > 2 SDs, symmetric	15	62	2–5 days, 6, 12 mo	No *P* values of statistical tests provided.	
	BW p3–p10, > 2 SDs, asymmetric	47	62	2–5 days, 6, 12 mo	No *P* values of statistical tests provided.	
Karagol (2019) (44)	BW < p10	55	200	1 mo	Left and right coronary artery diameters decreased in FGR.	No nonsignificant differences.
				6 mo	Left and right coronary artery diameters decreased in FGR.	No nonsignificant differences.
Kumar (2019) (45)	BW < 2SDs	35	35	*Day 1*	Cardiac morphology: LVIDd and LVIDs index increased in FGR. Diastolic function: TV E wave deceleration time decreased in FGR.	Systolic function: Fractional shortening, LVEF. Diastolic function: MV E, MV A, MV E/A, TV E, TV A, TV E/A, MV E wave deceleration time.
				*Day 2*	Cardiac morphology: LVIDd, LVIDs increased in FGR. Diastolic function: MV E, MV A, TV A decreased in FGR.	Systolic function: Fractional shortening, LVEF. Diastolic function: MV E/A, TV E, TV E/A, MV E wave deceleration time, TV E wave deceleration time.
				*Day 3*	Cardiac morphology: LVIDd and LVIDs index increased in FGR.	Systolic function: Fractional shortening, LVEF. Diastolic function: MV E, MV A, MV E/A, TV A, TV E/A, MV E wave deceleration time, TV E wave deceleration time.
Levent (2009) (46)	BW < p10	32	31	8.8 yr	No significant differences.	Cardiac morphology: LVEDd, IVSd, LVPWd, LVM, LVMI. Systolic function: LVEF, fractional shortening.
Mäkikallio (2016) (47)	BW < p10 and/or an UA PI > 2 SD	60	52	1 wk	No results of statistical tests between FGR and controls shown (only over time difference provided within group).	
				6 mo	No results of statistical tests between FGR and controls shown (only over time difference provided within group).	
Mikkola (2007) (48)	BW < 2 SDs	22	25	5 yr	No significant differences.	Cardiac morphology: IVSd, LVEDd, LV systolic diameter, LVPWd. Systolic function: Fractional shortening, LVEF, left ventricle stroke index, left ventricle cardiac index s.
Miyamoto (2013) (49)	BW < p10	14	23	2.8 h	Cardiac morphology: LVPWs decreased in FGR.	Cardiac morphology: LVESd, LVEDd, IVSs. Systolic function: Fractional shortening, LVEF. Diastolic function: Transmitral E, transmitral A, transmitral E/A, Ew, Aw, Ew/Aw, E/Ew.
Montaldo (2022) (50)	BW < p10 with FGR defined as EFW < p3 or < p10 combined with abnormal MCA, UA or uterine artery doppler arteries and/or abnormal CPR	105	105	6 h	Systolic function: LV cardiac output decreased in FGR.	Systolic function: RV cardiac output.
				24 h	Systolic function: LV cardiac output decreased in FGR.	Systolic function: RV cardiac output.
				48 h	No significant differences.	Systolic function: LV cardiac output, RV cardiac output.
				72 h	No significant differences.	Systolic function: RV cardiac output.
Morsing (2014) (51)	BW *z*-score < 2, being delivered on fetal indication by cesarean section	32	32	7 yr	No significant differences.	Cardiac morphology: LVMI, IVSd, LVPWd, LVEDd. Systolic function: Fractional shortening, LVEF. Diastolic function: Mitral E/A ratio, mitral velocity time interval, E wave deceleration time.
Niewiadomska–Jarosik (2016) (52)	BW < p10 with IUGR features	77	30	7.6 yr	Diastolic function: E wave, E/A ratio decreased in FGR. A wave, IVRT, deceleration time increased in FGR.	Cardiac morphology: LVEDd, IVSd, LVPWd. Systolic function: LVEF, fractional shortening.
Olander (2022) (53)	BW < 2 SD, < p3	23	48	5.8 yr	Diastolic function: Mitral E wave, A wave, septal E/E′ increased in FGR. Systolic function: MAPSE, TAPSE decreased in FGR.	Cardiac morphology: LV basal sphericity index, LV midpapillary sphericity index, RV base sphericity index, RV midcavity sphericity index. Diastolic function: Mitral E/A ratio, septal E′-wave peak velocity, septal E/E′ ratio. Systolic function: LVEF.
Olander (2020) (54)	BW < 2 SDs	39	90	34 h (AGA), 56 h (SGA)	Cardiac morphology: IVS increased in SGA. RV midcavity dimension decreased in SGA. Diastolic function: E wave, E/E′ decreased in SGA.	Cardiac morphology: Left atrial area, LV basal dimension, LVEDd, LVPWd, LV area, LVM, LV volume, right atrial area, RV basal dimension, RV area, LV basal sphericity index, LV midcavity sphericity index, RV basal sphericity index, RV midcavity sphericity index. Systolic function: LVEF. Diastolic function: MV lateral E′ wave peak velocity.
Patey (2019) (55)	EFW < p10 with Doppler evidence of placental dysfunction	33	54	0.5 days	Cardiac morphology: LVEDd decreased in FGR. RV/LVEDd ratio, IVS increased in FGR. Systolic function: RV cardiac output, IVS-S′, LV IVCT′, RV IVCT′ increased in FGR. Diastolic function: LV E/A, RV E/A, LV E′/A′, IVS E′/A′ decreased in FGR. LV IVRT′, RV IVRT′ increased in FGR.	Cardiac morphology: RVEDd. Systolic function: RV cardiac output, RV S′. Diastolic function: LV-E/E′.
Rodriguez–Guerineau (2018) (56)	BW < p10	25	25	3.5	Cardiac morphology: LV sphericity index, LV base-to-apex length, RV sphericity index, RV base-to-apex length, IVS, LVPWT, LVM decreased in FGR. Systolic function: LV stroke volume increased in FGR. MAPSE, lateral mitral annulus s′, septal mitral annulus s′, TAPSE, tricuspid annulus IVCT′ decreased in FGR. Diastolic function: Septal mitral annulus e′, tricuspid annulus e′, tricuspid annulus IVRT′ decreased in FGR.	Cardiac morphology: LV basal diameter, RV basal diameter, RWT. Systolic function: LV cardiac output, fractional shortening, RV stroke volume, RV cardiac output, tricuspid annulus s′, septal mitral annulus IVCT′, lateral mitral annulus IVCT′, lateral mitral annulus ET′. tricuspid annulus ET′. septal mitral annulus ET′. Diastolic function: Lateral mitral annulus e′, lateral mitral annulus a′, septal mitral annulus a′, LV E/A ratio, E mitral deceleration time, tricuspid annulus a′, RV E/A ratio, E tricuspid deceleration time, lateral mitral annulus IVRT′, septal mitral annulus IVRT′.
Rodriguez–Lopez (2023) (57)Male	BW < p10	108	142	26–36 yr	Cardiac morphology: LV basal diameter increased. Systolic function: LV MAPSE decreased.	Cardiac morphology: LV base-to-apex length, LV sphericity index, LV RWT, LA area, RV basal diameter, RV base-to-apex length, RV sphericity index, RV area. Systolic function: LVEF, LV cardiac output, mitral lateral annular peak systolic velocity, LV global longitudinal strain, fractional area change, TAPSE, tricuspid annular peak systolic velocity. Diastolic function: Mitral E/A, isovolumic relaxation time, tricuspid E/A.
Rodriguez-Lopez (2023) (57)Female	BW < p10	153	120	26–36 yr	Cardiac morphology: LV basal diameter, LV base-to-apex length, RV base-to-apex length increased. Systolic function: LVEF, MAPSE, mitral lateral annular peak systolic velocity, tricuspid annular peak systolic velocity decreased.	Cardiac morphology: LV sphericity index, LV RWT, LV area, RV basal diameter, RV sphericity index, RV area. Systolic function: LV cardiac output, LV global longitudinal strain, fractional area change, TAPSE. Diastolic function: Mitral E/A, isovolumic relaxation time, tricuspid E/A.
Sarvari (2017) (58)	BW < p10	58	96	10.75 yr	Cardiac morphology: LV and RV base to apex length, LV and RV sphericity index decreased in FGR. LV basal diameter increased in FGR. Systolic function: Mitral s′, LV global longitudinal strain decreased in FGR. Diastolic function: LV IVRT, RV IVRT increased in FGR.	Cardiac morphology: RWT, RV basal diameter, left atrial area, right atrial area. Systolic function: LV cardiac output, LVEF, tricuspid s′. Diastolic function: Mitral e′, tricuspid E/A ratio, tricuspid e′.
Sehgal (2018) (59)	BW or EFW < p10	20	20	10.4 days	No results of statistical tests between FGR and controls shown (only correlations between arterial mechanics and cardiac function parameters).	
Sehgal (2017) (60)	BW < p10	20	20	10.4 days	Systolic function: MAPSE decreased in FGR. Diastolic function: E wave, A wave, TDI mitral E′, TDI mitral A′ decreased in FGR. E/A ratio, TDI E′/A′, E/E′ increased in FGR.	Cardiac morphology: RWT. Systolic function: Fractional shortening. Diastolic function: TDI E′/A′
Sehgal (2013) (61)	BW < p3	20	20	76.5 h	Systolic function: Fractional shortening, LV cardiac output index decreased in SGA. Diastolic function: E wave, A wave, IVRT increased in SGA. E/A ratio, E deceleration time, IVRT increased in FGR.	No nonsignificant differences.
Sehgal (2013) (14)	BW < p3	20	20	76.5 h	Systolic function: Left ventricular global longitudinal strain, basal septal strain, basal septal SR, middle septal SR, apical septal strain, apical septal SR, basal lateral strain, basal lateral SR, middle lateral strain, middle lateral SR, apical lateral strain, apical lateral SR decreased in SGA.	No nonsignificant differences.
Sehgal (2022) (62)	BW < p10 with absent/reversed antenatal umbilical artery Doppler	28	26	10.1 days	Cardiac morphology: LVEDd, LVPWd, LVM increased in FGR. Diastolic function: Transmitral E/A increased in FGR.	Systolic function: Fractional shortening, LV cardiac output.
Suciu (2022) (63)	EFW < p10	18	18	1 day	Systolic function: RV cardiac output, TAPSE decreased in SGA. LVEF increased in SGA. Diastolic function: Tricuspid E/A decreased, mitral E, mitral A in SGA. IVRT increased in SGA.	Systolic function: Fractional shortening, LVEF, LV cardiac output. Diastolic function: Tricuspid E, tricuspid A.
				2 days	Systolic function: RV cardiac output, TAPSE decreased in SGA. LVEF increased in SGA. Diastolic function: Tricuspid E/A decreased, mitral E, mitral A in SGA. IVRT increased in SGA.	Systolic function: Fractional shortening, LVEF, LV cardiac output. Diastolic function: Tricuspid E, tricuspid A.
Vats (2021) (64)	BW < p10	100	100	53 h	Cardiac morphology: IVSs decreased in SGA. Systolic function: LV ET and RV ET decreased in SGA. RV IVCT increased in SGA. Diastolic function: RV IVRT increased in SGA.	Cardiac morphology: IVSd, LVIDd, LVIDs, LVPWd, LVPWs. Systolic function: LV IVCT. Diastolic function: E wave, A wave, E/A ratio, LV IVRT.
Verma (2023) (65)	BW < p3, or BW < p10 with FGR defined plus abnormal Doppler	24	30	24–48 h	Systolic function: TAPSE decreased in FGR.	Systolic function: Fractional shortening, LVEF, LV output, RV output.
				48 h	Systolic function: Fractional shortening, LV EF, TAPSE, RV cardiac output decreased in FGR.	Systolic function: LV output.
Yiallourou (2017) (66)	EFW < p10 plus absent or reversed EDF of UA	18	15	9 yr	No significant differences.	Cardiac morphology: LVIDd, LVIDs, LVPWd, sphericity index, RWT, left atrium. Systolic function: Stroke volume, cardiac output, LVEF, fractional shortening. Diastolic function: Mitral E wave velocity, mitral A wave velocity, mitral E/A ratio, left IVRT.
Zaharie (2019) (67)	EFW < p10 abnormal Doppler of the UA or/and MCA	40	21	1 and 6 mo	No results of statistical tests between FGR and controls shown (only over time difference provided within FGR group).	

A, diastolic late peak velocity; AGA, appropriate for gestational age; BW, birth weight; MCA, middle cerebral artery; con, control; E, diastolic early peak velocity; EDL, end-diastolic length; EF, ejection fraction; EFW, estimated fetal weight; Em, early myocardial peak velocity; ET, ejection time; FGR, fetal growth restriction; GA, gestational age; IVCT, isovolumetric contraction time; IVS, interventricular septum thickness; IVRT, isovolumetric relaxation time; LV, left ventricle; LVEDd, left ventricular end-diastolic diameter; LVEDs, left ventricular end-systolic diameter; LVEDdI, left ventricular end-diastolic diameter index; LVIDd, left ventricle internal diameter during diastole; LVIDs, left ventricle internal diameter during systole; LVM, left ventricular mass; LVMI, left ventricular mass index; LVPWd, left ventricular posterior wall thickness at end diastole; LVPWs, left ventricular posterior wall thickness at end systole; MAPSE, mitral annular plane systolic excursion; MV, mitral valve; p, percentile; RV, right ventricle; RVEDd, right ventricular end-diastolic diameter; RWT, relative wall thickness; SGA, small for gestational age; SL, strain longitudinal; TAPSE, tricuspid annular plane systolic excursion; TDI, tissue doppler imaging; UA, umbilical artery; VL, velocity longitudinal.

**Table 2.** Overview of the main study characteristics and results of included animal studies

Author (Year)	Species (Strain)	FGR Model	FGR, *n*	Con, *n*	Age	Significant Differences	Nonsignificant Results
Alsaied (2017) (68)	Mice (C57BL/6J)	Uterine artery ligation	12	12	12 wk	Cardiac morphology*:* LVIDs increased in FGR. Systolic function: Fractional shortening, LV EF decreased in FGR.	Cardiac morphology*:* IVSd, IVSs, LVPWd, LVPWs, LVIDd, LV mass.
					24 wk	Cardiac morphology: LVIDs increased in FGR. Systolic function: Fractional shortening, LV EF decreased in FGR	Cardiac morphology: IVSd, IVSs, LVPWd, LVPWs, LVIDd, LV mass.
					32 wk	Cardiac morphology: LVIDs increased in FGR. Systolic function: Fractional shortening, LV EF decreased in FGR.	Cardiac morphology: IVSd, IVSs, LVPWd, LVIDd, LV mass.
Coats (2021) (69)	Rat (SD)	Reduced uterine pressure perfusion	4	4	16 wk	Cardiac morphology: Stroke volume decreased in FGR. Systolic function: LV EF, cardiac output decreased in FGR.	Cardiac morphology: LV mass. Systolic function: Fractional shortening.
Kumar (2020) (70)	Rat(SD)	Hypoxic chamber	—	—	*Day 1*	No significant differences.	Cardiac morphology: LVEDd/weight, LVPWd/weight, stroke volume/weight, VCFc. Systolic function: Septal S′ velocity, LV LW S′ velocity. Diastolic function: MV E velocity, MV A velocity, LV LW E′ velocity, LV LW A′ velocity, LV LW E′/A′ velocity.
					*Day 3*	No significant differences.	Cardiac morphology*:* LVEDd/weight, LVPWd/weight, stroke volume/weight, VCFc. Systolic function*:* Septal S′ velocity, LV LW S′ velocity. Diastolic function*:* MV E velocity, MV A velocity, LV LW E′ velocity, LV LW A′ velocity, LV LW E′/A′ velocity.
					1 wk	Diastolic function: LV lateral wall E′/A′ decreased in FGR.	Cardiac morphology*:* LVEDd/weight, LVPWd/weight, stroke volume/weight, VCFc. Systolic function: Septal S′ velocity, LV LW S′ velocity. Diastolic function*:* MV E velocity, MV A velocity, LV LW E′ velocity, LV LW A′ velocity.
					2 wk	Systolic function: Septal S′, LV lateral wall increased in FGR (*P* < 0.008). Diastolic function: LV lateral wall E′, LV lateral wall A′ increased in FGR.	Cardiac morphology: LVEDd/weight, LVPWd/weight, stroke volume/weight. Diastolic function: MV E velocity, MV A velocity, LV LW E′/A′ velocity.
					4 wk	Diastolic function: LV lateral wall E′/A′ decreased in FGR.	Cardiac morphology: LVEDd/weight, LVPWd/weight, stroke volume/weight, VCFc. Systolic function: Septal S′ velocity, LV LW S′ velocity. Diastolic function: MV E velocity, MV A velocity, LV LW E′ velocity, LV LW A′ velocity.
					8 wk	Systolic function: LV lateral wall S′ increased in FGR. Diastolic function: MV E, LV lateral wall E′/A′ decreased in FGR. LV lateral wall A′ increased in FGR.	Cardiac morphology: LVEDd/weight, LVPWd/weight, stroke volume/weight, VCFc. Systolic function: Septal S′ velocity. Diastolic function: MV A velocity, LV LW E′ velocity.
Rueda-Clausen (2008) (71)	Rat, male(SD)	Hypoxic chamber	12	14	4 mo	No significant differences.	Cardiac morphology: RV diameter in diastole, IVSd, IVSs, LVIDd, LVIDs, LVPWTd, LVPWTs, M-mode trace LV end-diastolic volume. Systolic function: CO/weight, LV shortening fraction, LVEF. Diastolic function*:* Mitral E maximum velocity, Mitral A maximum velocity, Mitral E/A index, mitral deceleration time, MPI, Mitral IVRT, Mitral IVCT.
					12 mo	Cardiac morphology*:* RV diameter in diastole increased in FGR. IVSs, LVPWTs decreased in FGR. Diastolic function*:* Mitral E/A index, mitral IVRT increased in FGR. Mitral A maximum velocity, mitral E deceleration time decreased in FGR.	Cardiac morphology*:* IVSd, LVEDd, LVEDs, LVPWTd, M-mode trace LV end-diastolic volume. Systolic function: CO/weight, LV shortening fraction, LVEF. Diastolic function: Mitral A maximum velocity, Mitral IVCT.
	Rat, female(SD)	Hypoxic chamber	13	12	4 mo	No significant differences.	Cardiac morphology: RV diameter in diastole, IVSd, IVSs, LVIDd, LVIDs, LVPWTd, LVPWTs, M-mode trace LV end-diastolic volume. Systolic function: CO/weight, LV shortening fraction, LVEF. Diastolic function: Mitral E maximum velocity, Mitral A maximum velocity, Mitral E/A index, Mitral deceleration time, MPI index, Mitral IVRT, Mitral IVCT.
					12 mo	Diastolic function: Mitral A maximum velocity, mitral E deceleration time decreased in FGR. Mitral E/A index, mitral IVRT increased in FGR.	Cardiac morphology*:* IVSd, LVEDd, LVEDs, LVPWTd, M-mode trace LV end-diastolic volume. Systolic function: CO/weight, LV shortening fraction, LVEF. Diastolic function: Mitral E maximum velocity, Mitral IVCT.
Shah (2017) (72)	Rat, male(SD)	Hypoxic chamber	–	–	13 wk	No significant differences.	Cardiac morphology: LVAWd, LVAWs, LVPWTd, LVPWTs, LVIDd, LVIDs, LVEDV, LVESV. Systolic function: LVEF, fractional shortening. Diastolic function: Mitral E max velocity, Mitral A max velocity, Mitral E/A index, Mitral deceleration time, IVRT, Tei index, Mitral annular E′ velocity, Mitral annular A′ velocity, E/E′ action.
	Rat, female(SD)	Hypoxic chamber	–	–	13 wk	Diastolic function: Mitral A max velocity decreased in FGR. Mitral E deceleration time increased in FGR.	Cardiac morphology: LVAWd, LVAWs, LVPWTd, LVPWTs, LVIDd, LVIDs, LVEDV, LVESV. Systolic function: LVEF, fractional shortening.Diastolic function: Mitral E max velocity, Mitral E/A index, Mitral deceleration time, IVRT, Tei index, Mitral annular E′ velocity, Mitral annular A′ velocity, E/E′ ratio.
Thompson, 2018 (42)	Guinea pig, male	Hypoxic chamber	9	9	3 mo	Systolic function: Stroke volume, cardiac output, LV EF, fractional shortening decreased in FGR.	Cardiac morphology: LV diameter in diastole, LV diameter in systole, LVPWTd, LVPWTs, IVSd, IVSs.
	Guinea pig, female	Hypoxic chamber	9	9	3 mo	No significant differences.	Cardiac morphology: LV diameter in diastole, LV diameter in systole, LVPWTd, LVPWTs, IVSd, IVSs. Systolic function: Stroke volume, CO, LVEF, fractional shortening.

A, diastolic late peak velocity; con, control; E, diastolic early peak velocity; EF, ejection fraction; FGR, fetal growth restriction; IVRT, isovolumetric relaxation time; IVS, interventricular septum thickness; LV, left ventricle; LVIDd, left ventricle internal diameter during diastole; LVIDs, left ventricle internal diameter during systole; LVPWd, left ventricular posterior wall thickness at end diastole; LVPWs, left ventricular posterior wall thickness at end systole; NA, not applicable; MV, mitral valve; RV, right ventricle; SD, Sprague–Dawley, VCF, circumferential shortening velocity.

### Human Studies

#### Study characteristics of human studies.

All studies were either prospective cohort studies or case-control studies. Almost all studies defined the study population as SGA with birth weight below the 10th percentile for gestational age or minus two standard deviations corrected for gestational age, and only eight studies defined FGR more accurately with EFW and abnormal prenatal doppler measurements (36, 37, 47, 50, 55, 65–67). None of the studies reported the measurements per sex to assess sex-specific differences. The majority of cardiac ultrasounds occurred during the neonatal phase, with a range of 2.8 h after birth until 41 yr old. Only three studies stratified results based on severity of FGR (31, 32, 43) and one study stratified per sex (57). The gestational age at birth was mostly at term.

#### Effect of FGR on conventional echocardiographic parameters in human studies.

An overview of the echocardiographic outcomes is shown in Table 3, and the full data extraction is available in Supplemental Table S4. According to our predefined protocol, we were unable to perform a meta-analysis on all reported outcomes because of lack of more than five studies reporting on the same outcome at the similar age. The performed meta-analyses in human studies will be discussed below per age group.

**Table 3.** Summary overall effect derived from the forest plots per age category in human FGR versus control

	Neonate	Child
*Primary outcome measures*		
Cardiac morphology		
Left ventricular mass	↓	=
Left ventricular mass index	=	NA
Left ventricle end-diastolic dimension	=	=
Left ventricular posterior wall thickness in diastole	=	=
Interventricular septum thickness in diastole	↓	=
Systolic function		
Mitral annular systolic peak velocity	↓	=
Tricuspid annular systolic peak velocity	NA	=
Left ventricle cardiac output index	=	NA
Left ventricular ejection fraction	=	=
Fractional shortening	=	=
Diastolic function		
E/A ratio mitral valve	=	=
E/A ratio tricuspid valve	NA	=
Mitral lateral e	↓	=
*Secondary outcome measures*		
Heart rate	↑	**=**
Systolic blood pressure	↑	**=**
Diastolic blood pressure	↑	**=**

An upward pointing arrow (↑) represents a significant increase in FGR offspring, a downward pointing arrow (↓) represents a significant decrease in FGR offspring, and an equal to sign (=) represents no significant difference in outcome. A, late diastole; E, early diastole; NA, not applicable.

#### Meta-analysis of echocardiographic data in neonates.

Ultrasound measurement on cardiac morphology showed a smaller left ventricular mass with a SMD of −0.97 ([−1.73; −0.22], *P* = 0.03) in FGR neonates compared with controls (Supplemental Fig. S2*A*). However, note that the lower limit of the CI is very close to 0, and a very high between-study heterogeneity is present (I^2^ = 93%). Some of these studies also reported ventricular mass index to correct for body weight, which did not reveal a significant difference (Supplemental Fig. S2*B*). The left ventricular posterior wall thickness and end-diastolic dimension of the left ventricle were similar between groups (Supplemental Fig. S2, *C* and *D*). The interventricular septal thickness (IVS) during diastole was smaller in FGR than in controls with an SMD of −0.74 ([−1.25; −0.24], *P* = 0.00), and a very high between-study heterogeneity of I^2^ = 91% (Supplemental Fig. S2*E*).

Echocardiographic systolic function measurements showed a reduced mitral annular systolic peak velocity in FGR of −0.85 SMD ([−1.27; −0.44], *P* = 0.01, I^2^ = 68%) compared with controls (Supplemental Fig. S3*A*). However, left ventricular cardiac output index, left ventricular ejection fraction, and fractional shortening were similar between groups (Supplemental Fig. S3, *B–D*).

Echocardiographic diastolic function measurements showed a reduced mitral lateral velocity during early diastole (e) in FGR compared with controls (SMD −0.66 [−0.98; −0.33], *P* < 0.01, I^2^ = 39%, Supplemental Fig. S4*A*), but no difference in E/A ratio of the mitral valve was observed (Supplemental Fig. S4*B*).

Meta-analysis of secondary outcomes showed an increase in both systolic (SMD 0.83 [0.20; 1.45], *P* = 0.01) and diastolic blood pressure in FGR neonates (SMD 0.65 [0.21; 1.08], *P* < 0.01) compared with control neonates (Supplemental Fig. S5, *A* and *B*). Both meta-analyses showed very high between-study heterogeneity of I^2^ = 92% and I^2^ = 84%, respectively. Heart rate did not differ between the groups (Supplemental Fig. S5*C*).

#### Meta-analysis of echocardiographic data in children.

A total of 23 studies reported echocardiographic data in children, with ages ranging between 30 days and 10.1 yr old. Ultrasound measurements of cardiac morphology (Supplemental Fig. S6), systolic function (Supplemental Fig. S7), and diastolic function (Supplemental Fig. S8) were all similar between FGR and control.

In contrast to the neonatal data, meta-analysis on secondary outcomes, including systolic and diastolic blood pressure and heart rate, showed no differences between the groups (Supplemental Fig. S9).

#### Echocardiographic data in adults.

As only two studies were performed in the adult age category (age range, 20–49 yr old), we were not able to perform a meta-analysis on this data (26, 57). These two studies showed subtle changes in echocardiographic parameters and regarding cardiac morphology in particular (i.e., the heart of FGR offspring was slightly enlarged) (Table 1). One of these studies determined echocardiographic parameters for male and female offspring separately and showed that female offspring was more affected (57).

#### Effect of FGR on myocardial strain analysis using speckle tracking in humans: subtle (preclinical) changes of the heart.

Four studies investigated subclinical cardiac changes with the use of speckle-tracking ultrasound and three found impaired global longitudinal strain (12, 14, 23). One of these studies investigated longitudinal changes and reported no differences directly at birth between FGR and control, yet over a short time of aging of 3 to 4 mo, an impaired cardiac development was observed in FGR offspring compared with control offspring in left ventricular longitudinal strain (12). Another study showed that the severity of decrease of global longitudinal strain depended on the severity of FGR as measured at the age of 1 wk (23). Lastly, one study performed strain analysis in males and females separately, but no significant differences were observed in global longitudinal strain in both male and female offspring (57).

#### Risk of bias in human studies.

Quality assessment demonstrated a relatively low study quality, especially in the representativeness of the FGR group, because of restricted use of prenatal biometry and Doppler measurements to define FGR, but also in the outcome assessment because of missing reporting on blinding, and unclear reporting on loss of follow-up of participants in the cohort (Supplemental Fig. S10*A* and Supplemental Table S5). Funnel plots were not created according to protocol (<20 studies per outcome).

### Animal Studies

#### Study characteristics of animal studies.

All five animal studies included rodents with species varying from rats (*n* = 4) (70–72), mice (*n* = 1) (68), to guinea pigs (*n* = 1) (73) (Table 2). Surgical induction by uterine artery ligation was applied in the mice study and by reduced uterine perfusion pressure in one rat study while the other studies induced FGR by use of a hypoxic chamber. Three of the studies assessed sex-specific differences (71–73). Age ranged from 1 day old until 12 mo old. Three of the studies repeated the cardiac ultrasound parameters at multiple time points (68, 70, 71).

#### Effect of FGR on conventional echocardiographic parameters in animal studies.

An overview of the echocardiographic outcomes is shown in Table 2, and the full data extraction is available in Supplemental Table S4. Considering the restricted overlap in reported echocardiographic parameters, we were unable to perform a meta-analysis.

All five animal studies showed either systolic or diastolic dysfunction of the left ventricle (68, 70–73). Signs of systolic dysfunction included decreased ejection fraction and fractional shortening (68, 70, 73), while signs of diastolic dysfunction included reduced early and late peak velocity over the mitral valve and increased isovolumetric relaxation time (70–72). Cardiac morphological changes were observed with an increase in inner diameter of the left ventricle and decrease in septal thickness (68, 71). Two studies showed either persistence or progression of cardiac dysfunction over time (68, 70). Sex-specific differences were reported to the prejudice of male offspring in two studies (71, 73), however, another study only observed effects on outcome in female offspring (72).

Secondary outcomes reported in these six animal studies showed conflicting effects with no difference or decreased stroke volume (70, 73), no difference or transient decreased heart rate after birth (70, 71), and no difference or an increase in blood pressure in FGR offspring (71, 73). The included studies reporting on pulse wave velocity as a marker for aortic stiffness appeared persistently increased over time in the FGR group with the largest difference compared with control offspring direct after birth (70).

#### Risk of bias assessment in animal studies.

Quality assessment of animal studies revealed poor or absence of reporting of essential information, and consequently, we designated the risk of most types of bias largely as unclear (Supplemental Fig. S10*B* and Supplemental Table S6).

## DISCUSSION

Our meta-analysis revealed small differences in echocardiographic parameters between human FGR and control offspring in the neonatal period only, namely, a reduced left ventricular mass and interventricular septum thickness, reduced mitral annular peak velocity, and mitral lateral early diastolic velocity. The few clinical studies on myocardial strain analysis using speckle tracking unanimously showed small preclinical changes in longitudinal global strain FGR offspring. The limited number of animal studies per individual outcome prevented us from performing a meta-analysis.

### Age-Dependent Influence of FGR on Conventional Echocardiographic Parameters in Human Studies

Our meta-analysis of human studies revealed only differences in the neonatal population and not during childhood. Potential explanations are related to the small group sizes and the selection of studies as the age range of children in the included studies is skewed with most studies until 8 yr old, only one study at 11 yr old, and only two studies in the (young) adult age category. The limited number of studies in the adult age group showed subtle changes in echocardiographic parameters. Nevertheless, as the onset of some cardiovascular deteriorations (e.g., atherogenesis) occurs later during childhood, an effect of FGR would possibly be more apparent if more studies were performed within this age category (74).

Alternatively, the discrepancy between the neonatal and childhood comparison might reflect cardiac plasticity. The increased workload of the fetal heart as a response to hemodynamic redistribution during pregnancy possibly recovers since the birth of the neonate and thereby discontinued exposure to an adverse hypoxic environment allows rehabilitation. The observed difference in the neonatal study population might more purely reflect the FGR impact since the exposure to other factors has not occurred yet. Therefore, the diagnosis of FGR might just be as valuable to predict long-term outcomes as the neonatal echocardiographic parameters. Besides the natural ongoing maturation and growth of the heart as part of aging, other factors at older age such as catch-up growth, genetic resilience, or second hits unavoidably play a more significant role in cardiovascular risk development (4, 8, 75); these factors might outweigh the effect of FGR itself later in life.

Nevertheless, at all ages, the echocardiographic differences in FGR offspring could be considered an early sign of susceptibility to develop cardiovascular disease. Specifically, studies on myocardial strain analysis using speckle tracking showed impaired global strain in the left ventricle in FGR, which suggests preclinical left ventricular dysfunction. However, the relation to relevant long-term consequences remains to be elucidated. The same applies to the most optimal time frame to perform cardiac ultrasound to detect patients with FGR at risk.

### Echocardiographic Parameters in Animal Models of FGR: A Small Number of Studies and Use of Small Animals Only

Our systematic review identified a limited number of studies on the effect of FGR on echocardiographic parameters in animals. This may be because ultrasound is noninvasive in humans, while in rodents it requires anesthesia, making humans the preferred study population. In animals, anesthesia can affect echocardiographic measurements, which may explain the conflicting observations on some parameters (76). Our systematic review mostly included studies using a hypoxic chamber to induce FGR, while the surgical induction by uterine artery ligations appears to be more suitable to study cardiac function in FGR (77). Currently, none of the studies used larger animals such as pigs or sheep. The use of large animal models of FGR could increase the translational value (78) as they are anatomically closer to humans, and cardiac ultrasound can be performed in these animals without anesthesia.

The few studies that have been performed in animal models all showed some differences in systolic or diastolic function in the FGR group. In contrast to human studies, the effect seems to either persist or become greater at older age. This might be explained by the accelerated aging observed in animals while the human studies only included young adults as long-term follow-up remains challenging in human trials. On the other hand, the included animal studies were of poor quality, which is associated with an overestimation of intervention effects.

### Strengths and Limitations

Our systematic review and meta-analysis are the first to provide an overview of all the cardiac ultrasound measurements available (no restrictions applied on species or specific measurements) in FGR offspring in both human and animal studies, which allows for evaluation of modifying factors and highlights the need of uniformization of study design and improvement of study quality. With that, we aimed to create awareness of the limitations of included studies among researchers in the field and the results of this study could be used to optimize future study designs on this topic.

Although intended, our meta-analysis is limited to being unable to study the influence of several potential factors because of underreporting and small group sizes. Sex is believed to be of great influence from the developmental origin of disease perspective and has also been described in a few animal studies (71–73), thereupon it is unfortunate that the exact impact of sex remains unclear as we were unable to perform meta-regression. Only one human study investigated sex-specific differences in echocardiographic parameters including myocardial strain in which female SGA offspring appeared to be more susceptible to cardiac dysfunction (57). In addition, interobserver and intraobserver differences are a well-known limitation of ultrasound. Although we are aware that this hampers reproducibility, we were unable to take this into account and therefore unable to distinguish the influence on heterogeneity of our data. In general, there were too limited data to perform subgroup analyses, leaving considerable levels of between-study heterogeneity unexplained for a number of outcomes. In addition, the interdependence of multiple ultrasound outcomes increases the statistical chance of false-positive findings. We acknowledge that this poses interpretive challenges to the results of our meta-analyses, and further research is needed to elucidate the sources of heterogeneity in these analyses. Of note, the poor reporting of key quality indicators and measures to reduce bias in both human and animal studies reduces the confidence we can have in their findings and should be taken into account when interpreting the results of our data synthesis. The reader should also be aware that the limited number of studies included in our meta-analyses prevented us from assessing publication bias, and we can therefore not rule out an influence of publication bias on our meta-analysis. Finally, our meta-analysis focused on cardiac ultrasound measurements, rather than potential adverse arterioventricular interactions in the FGR group. FGR has an established negative impact on vascular development and maturation, which can increase cardiac afterload (79). This can trigger a cascade of adverse arterioventricular interactions that ultimately lead to cardiac dysfunction (80). It is tempting to speculate that the age-dependent influence of FGR on cardiac parameters is explained by normalization of the cardiac afterload during childhood. The interaction between the vascular system and cardiac performance was not the focus of this meta-analysis, however. Further studies are needed to elucidate mechanisms underlying the normalization of cardiac parameters in FGR children during childhood.

### Future Perspectives

Although cardiac ultrasound offers a noninvasive manner to detect or monitor early signs of cardiovascular predisposition in FGR offspring, the clinical value of echocardiography alone to reflect a child’s vascular status appears limited and hampers further implementation in clinical practice. The largest challenges include identification of optimal timing and the exact relation to long-term cardiovascular function. Besides, most likely one specific ultrasound measurement will not form a good predictor, but rather the combination of multiple promising echocardiographic or the combination of other parameters could contribute to the estimation of an individual cardiovascular risk profile. Regarding echocardiographic parameters, we would recommend focusing more on myocardial strain analysis and include an older human study population or larger animal models of FGR. Other imaging parameters that hold promise involve cardiac MRI to evaluate cardiac remodeling in FGR (81), while nonimaging parameters such as arterial stiffness measured with carotid distensibility coefficient or pulse wave velocity might offer a better risk estimation (23). As the best prevention of cardiovascular disease so far concerns hypertensive treatment, conventional blood pressure measurements over time to start early therapy in FGR offspring might provide the most gainful approach (82).

## DATA AVAILABILITY

All data are provided within the article or supplemental data.

## SUPPLEMENTAL DATA

10.6084/m9.figshare.25479805.v2 Tables S1–S6 and Supplemental Figs. S1–S10: https://doi.org/10.6084/m9.figshare.25479805.v2.

## GRANTS

This study was funded by ZonMw, MKMD Synthesis of Evidence, Grant 114024183 (to F.T.) and UMC Utrecht, Department of Pediatrics, Clinical Research Fellowship 2023 (to F.T.).

## DISCLOSURES

None of the other authors has any conflicts of interest, financial or otherwise, to disclose.

## AUTHOR CONTRIBUTIONS

M.v.d.M., S.C.A.J., K.E.W., A.T.L., and F.T. conceived and designed research; M.v.d.M. and F.T. analyzed data; M.v.d.M., K.T.N., S.C.A.J., J.K., H.S.S., K.E.W., A.T.L., and F.T. interpreted results of experiments; M.v.d.M. and F.T. prepared figures; M.v.d.M. and F.T. drafted manuscript; M.v.d.M., K.T.N., J.K., H.S.S., K.E.W., A.T.L., and F.T. edited and revised manuscript; F.T. approved final version of manuscript.

## References

[1] Roth GA, Mensah GA, Johnson CO, Addolorato G, Ammirati E, Baddour LM, , et al Global Burden of Cardiovascular Diseases and Risk Factors, 1990-2019: Update From the GBD 2019 Study. J Am Coll Cardiol 76: 2982–3021, 2020 [Erratum in J Am Coll Cardiol 77: 1958–1959, 2021]. doi:10.1016/j.jacc.2020.11.010. 33309175 PMC7755038

[2] Barker DJ, Osmond C, Golding J, Kuh D, Wadsworth ME. Growth in utero, blood pressure in childhood and adult life, and mortality from cardiovascular disease. BMJ 298: 564–567, 1989. doi:10.1136/bmj.298.6673.564. 2495113 PMC1835925

[3] Visentin S, Grumolato F, Nardelli GB, Di Camillo B, Grisan E, Cosmi E. Early origins of adult disease: low birth weight and vascular remodeling. Atherosclerosis 237: 391–399, 2014. doi:10.1016/j.atherosclerosis.2014.09.027. 25463063

[4] Youssef L, Castellani R, Valenzuela‐Alcaraz B, Sepulveda‐Martinez Á, Crovetto F, Crispi F. Cardiac remodeling from the fetus to adulthood. J Clin Ultrasound 51: 249–264, 2023. doi:10.1002/jcu.23336. 36785500

[5] Karmali KN, Lloyd-Jones DM. Achieving and maintaining cardiovascular health across the lifespan. Curr Epidemiol Rep 1: 75–81, 2014. doi:10.1007/s40471-014-0011-7.

[6] Malhotra A, Allison BJ, Castillo-Melendez M, Jenkin G, Polglase GR, Miller SL. Neonatal morbidities of fetal growth restriction: pathophysiology and impact. Front Endocrinol (Lausanne) 10: 55, 2019. doi:10.3389/fendo.2019.00055. 30792696 PMC6374308

[7] Kamphof HD, Posthuma S, Gordijn SJ, Ganzevoort W. Fetal growth restriction: mechanisms, epidemiology, and management. Maternal-Fetal Medicine 4: 186–196, 2022. doi:10.1097/FM9.0000000000000161. PMC12094350 40406022

[8] Crispi F, Miranda J, Gratacós E. Long-term cardiovascular consequences of fetal growth restriction: biology, clinical implications, and opportunities for prevention of adult disease. Am J Obstet Gynecol 218: S869–S879, 2018. doi:10.1016/j.ajog.2017.12.012. 29422215

[9] Crispi F, Crovetto F, Rodriguez-López M, Sepúlveda-Martinez Á, Miranda J, Gratacós E. Postnatal persistence of cardiac remodeling and dysfunction in late fetal growth restriction. Minerva Obstet Gynecol 73: 471–481, 2021. doi:10.23736/S2724-606X.21.04823-5. 33949826

[10] Cruz-Lemini M, Crispi F, Valenzuela-Alcaraz B, Figueras F, Sitges M, Bijnens B, Gratacós E. Fetal cardiovascular remodeling persists at 6 months in infants with intrauterine growth restriction. Ultrasound Obstet Gynecol 48: 349–356, 2016. doi:10.1002/uog.15767. 26415719

[11] Lloyd-Jones DM, Hong Y, Labarthe D, Mozaffarian D, Appel LJ, Van Horn L, Greenlund K, Daniels S, Nichol G, Tomaselli GF, Arnett DK, Fonarow GC, Ho PM, Lauer MS, Masoudi FA, Robertson RM, Roger V, Schwamm LH, Sorlie P, Yancy CW, Rosamond WD; American Heart Association Strategic Planning Task Force and Statistics Committee. Defining and setting national goals for cardiovascular health promotion and disease reduction: The American Heart Association’s strategic impact goal through 2020 and beyond. Circulation 121: 586–613, 2010. doi:10.1161/CIRCULATIONAHA.109.192703. 20089546

[12] Änghagen O, Engvall J, Gottvall T, Nelson N, Nylander E, Bang P. Developmental differences in left ventricular strain in IUGR vs. control children the first three months of life. Pediatr Cardiol 43: 1286–1297, 2022. doi:10.1007/s00246-022-02850-y. 35333947 PMC9293814

[13] Akazawa Y, Hachiya A, Yamazaki S, Kawasaki Y, Nakamura C, Takeuchi Y, Kusakari M, Miyosawa Y, Kamiya M, Motoki N, Koike K, Nakamura T. Cardiovascular remodeling and dysfunction across a range of growth restriction severity in small for gestational age infants– implications for fetal programming. Circ J 80: 2212–2220, 2016. doi:10.1253/circj.CJ-16-0352. 27535477

[14] Sehgal A, Doctor T, Menahem S. Cardiac function and arterial indices in infants born small for gestational age: analysis by speckle tracking. Acta Paediatr 103: e49–e54, 2014. doi:10.1111/apa.12465. 24127769

[15] Page MJ, McKenzie JE, Bossuyt PM, Boutron I, Hoffmann TC, Mulrow CD, Shamseer L, Tetzlaff JM, Akl EA, Brennan SE, Chou R, Glanville J, Grimshaw JM, Hróbjartsson A, Lalu MM, Li T, Loder EW, Mayo-Wilson E, McDonald S, McGuinness LA, Stewart LA, Thomas J, Tricco AC, Welch VA, Whiting P, Moher D. The PRISMA 2020 statement: an updated guideline for reporting systematic reviews. *BMJ* 29:372: n71, 2021. doi:10.1136/bmj.n71. PMC8005924 33782057

[16] Gillis EE, Williams JM, Garrett MR, Mooney JN, Sasser JM. The Dahl salt-sensitive rat is a spontaneous model of superimposed preeclampsia. Am J Physiol Regul Integr Comp Physiol 309: R62–R70, 2015. doi:10.1152/ajpregu.00377.2014. 25904684 PMC4491533

[17] Sharkey LC, McCune SA, Yuan O, Lange C, Fray J. Spontaneous pregnancy-induced hypertension and intrauterine growth restriction in rats. Am J Hypertens 14: 1058–1066, 2001. doi:10.1016/s0895-7061(01)02161-6. 11710786

[18] Swanson AM, David AL. Animal models of fetal growth restriction: considerations for translational medicine. Placenta 36: 623–630, 2015. doi:10.1016/j.placenta.2015.03.003. 25819810

[19] Hooijmans CR, Rovers MM, De Vries RBM, Leenaars M, Ritskes-Hoitinga M, Langendam MW. SYRCLE’s risk of bias tool for animal studies. BMC Med Res Methodol 14: 43–49, 2014. doi:10.1186/1471-2288-14-43. 24667063 PMC4230647

[20] Wells G, Shea B, O’Connell D, Peterson J, Welch V, Losos M, Tugwell P. The Newcastle-Ottawa Scale (NOS) for assessing the quality of non-randomised studies in meta-analyse.

[21] Higgins JPT, Thomas J, Chandler J, Cumpston M, Li T, Page MJ, Welch VA **(editors).** Cochrane Handbook for Systematic Reviews of Interventions. Chichester, UK: John Wiley & Sons, 2019.

[22] Aburawi EH, Malcus P, Thuring A, Fellman V, Pesonen E. Coronary flow in neonates with impaired intrauterine growth. J Am Soc Echocardiogr 25: 313–318, 2012. doi:10.1016/j.echo.2011.11.019. 22177908

[23] Akazawa Y, Hachiya A, Yamazaki S, Kawasaki Y, Nakamura C, Takeuchi Y, Kusakari M, Miyosawa Y, Kamiya M, Motoki N, Koike K, Nakamura T. Cardiovascular remodeling and dysfunction across a range of growth restriction severity in small for gestational age infants- implications for fetal programming. Circ J 80: 2212–2220, 2016. doi:10.1253/circj.CJ-16-0352. 27535477

[24] Akazawa Y, Yamazaki S, Obinata H, Hachiya A, Kamiya M, Motoki N, Nakamura T. Decreased circulating insulin-like growth factor 1 levels are associated with cardiac diastolic dysfunction in small for gestational age infants. Am J Perinatol 35: 1178–1185, 2018. doi:10.1055/s-0038-1642060. 29689577

[25] Altın H, Karaarslan S, Karataş Z, Alp H, Şap F, Baysal T. Evaluation of cardiac functions in term small for gestational age newborns with mild growth retardation: a serial conventional and tissue Doppler imaging echocardiographic study. Early Hum Dev 88: 757–764, 2012. doi:10.1016/j.earlhumdev.2012.04.003. 22591553

[26] Arnott C, Skilton MR, Ruohonen S, Juonala M, Viikari JSA, Kähönen M, Lehtimäki T, Laitinen T, Celermajer DS, Raitakari OT. Subtle increases in heart size persist into adulthood in growth restricted babies: the Cardiovascular Risk in Young Finns Study. Open Heart 2: e000265, 2015. doi:10.1136/openhrt-2015-000265. 26339495 PMC4555072

[27] Bjarnegård N, Morsing E, Cinthio M, Länne T, Brodszki J. Cardiovascular function in adulthood following intrauterine growth restriction with abnormal fetal blood flow. Ultrasound Obstet Gynecol 41: 177–184, 2013. doi:10.1002/uog.12314. 23023990

[28] Castagno M, Menegon V, Monzani A, Zanetta S, Secco GG, Rosso R, Binotti M, Maiuri L, Di Mario C, Gazzolo D, Ferrero F, Genoni G. Small-for-gestational-age birth is linked to cardiovascular dysfunction in early childhood. Am Heart J 217: 84–93, 2019. doi:10.1016/j.ahj.2019.08.004. 31520898

[29] Cinar B, Sert A, Gokmen Z, Aypar E, Aslan E, Odabas D. Left ventricular dimensions, systolic functions, and mass in term neonates with symmetric and asymmetric intrauterine growth restriction. Cardiol Young 25: 301–307, 2015. doi:10.1017/S1047951113002199. 24355690

[30] Cohen E, Whatley C, Wong FY, Wallace EM, Mockler JC, Odoi A, Hollis S, Horne RSC, Yiallourou SR. Effects of foetal growth restriction and preterm birth on cardiac morphology and function during infancy. Acta Paediatr 107: 450–455, 2018. doi:10.1111/apa.14144. 29115693

[31] Crispi F, Bijnens B, Figueras F, Bartrons J, Eixarch E, Le Noble F, Ahmed A, Gratacos E. Fetal growth restriction results in remodeled and less efficient hearts in children. Circulation 121: 2427–2436, 2010. doi:10.1161/CIRCULATIONAHA.110.937995. 20497977

[32] Crispi F, Figueras F, Cruz-Lemini M, Bartrons J, Bijnens B, Gratacos E. Cardiovascular programming in children born small for gestational age and relationship with prenatal signs of severity. Am J Obstet Gynecol 207: 121.e1–121.e9, 2012. doi:10.1016/j.ajog.2012.05.011. 22717268

[33] Czernik C, Rhode S, Metze B, Bührer C, Schmitz L. Comparison of left ventricular cardiac dimensions between small and appropriate for gestational age preterm infants below 30 weeks of gestation. J Perinat Med 41: 219–226, 2013. doi:10.1515/jpm-2012-0032. 23093303

[34] Elmakaty I, Amarah A, Henry M, Chhabra M, Hoang D, Suk D, Ron N, Dygulska B, Sy F, Gudavalli MB, Nadroo AM, Narula P, Gad A. Perinatal factors impacting echocardiographic left ventricular measurement in small for gestational age infants: a prospective cohort study. BMC Pediatr 23: 393, 2023. doi:10.1186/s12887-023-04204-w. 37553638 PMC10411023

[35] Faienza MF, Brunetti G, Delvecchio M, Zito A, De Palma F, Cortese F, Nitti A, Massari E, Gesualdo M, Ricci G, Carbonara S, Giordano P, Cavallo L, Scicchitano P, Ciccone MM. Vascular function and myocardial performance indices in children born small for gestational age. Circ J 80: 958–963, 2016. doi:10.1253/circj.CJ-15-1038. 26861187

[36] Fouzas S, Karatza AA, Davlouros PA, Chrysis D, Alexopoulos D, Mantagos S, Dimitriou G. Neonatal cardiac dysfunction in intrauterine growth restriction. Pediatr Res 75: 651–657, 2014. doi:10.1038/pr.2014.22. 24522102

[37] Fouzas S, Karatza AA, Davlouros PA, Chrysis D, Alexopoulos D, Mantagos S, Dimitriou G. Heterogeneity of ventricular repolarization in newborns with intrauterine growth restriction. Early Hum Dev 90: 857–862, 2014. doi:10.1016/j.earlhumdev.2014.09.009. 25463832

[38] Garofoli F, Mannarino S, Montanari L, Cerbo R, Tzialla C, Mazzucchelli I, Angelini M, Codazzi AC, Mongini ME, Manzoni P, Tinelli C, Spinillo A, Stronati M. Variation of B-type natriuretic peptide concentrations and intrauterine growth restriction: mother, fetus and newborn. J Biol Regul Homeost Agents 26: 733–739, 2012. 23241123

[39] Guajardo CD, Mandelbaum V, Linderkamp O. Cardiac output and cerebral blood flow velocity in small for gestational age infants during the first 5 days after birth. Early Hum Dev 37: 187–193, 1994. doi:10.1016/0378-3782(94)90078-7. 7925077

[40] Gürses D, Seyhan B. Evaluation of cardiac systolic and diastolic functions in small for gestational age babies during the first months of life: a prospective follow-up study. Cardiol Young 23: 597–605, 2013. doi:10.1017/S1047951112001679. 23402323

[41] Harada K, Suzuki T, Takahashi Y, Ito T, Toyono M, Ishida A, Takada G. Abnormal left ventricular diastolic filling patterns in small-for-gestational-age infants. Early Hum Dev 51: 197–204, 1998. doi:10.1016/s0378-3782(97)00122-9. 9692789

[42] Ishii H, Takami T, Fujioka T, Mizukaki N, Kondo A, Sunohara D, Hoshika A, Akutagawa O, Isaka K. Comparison of changes in cerebral and systemic perfusion between appropriate- and small-for-gestational-age infants during the first three days after birth. Brain Dev 36: 380–387, 2014. doi:10.1016/j.braindev.2013.06.006. 23838311

[43] Ivanov DO, Saint Petersburg State Pediatric Medical University, Saint Petersburg, Russia, Derevtsov VV. Peculiarities of heart functional state in children born with different types of intrauterine growth retardation of mild degrees in the first year of life. Pediatria Journal Pediatria 99: 70–75, 2020. doi:10.24110/0031-403X-2020-99-1-70-75.

[44] Karagol BS, Kundak AA, Örün UA. Comparison of the diameter of coronary arteries between small for gestational age (SGA) and appropriate for gestational age (AGA) newborn infants. J Matern Fetal Neonatal Med 34: 907–912, 2021. doi:10.1080/14767058.2019.1622668. 31113241

[45] Kumar M, Santhanam S, Thomas N, Jana AK. A prospective observational study comparing cardiac function of small for gestational age with appropriate for gestational age babies using serial echocardiographic studies. J Matern Fetal Neonatal Med 32: 2194–2199, 2019. doi:10.1080/14767058.2018.1429392. 29338497

[46] Levent E, Atik T, Darcan S, Ulger Z, Gökşen D, Ozyürek AR. The relation of arterial stiffness with intrauterine growth retardation. Pediatr Int 51: 807–811, 2009. doi:10.1111/j.1442-200X.2009.02905.x. 19508453

[47] Mäkikallio K, Shah J, Slorach C, Qin H, Kingdom J, Keating S, Kelly E, Manlhiot C, Redington A, Jaeggi E. Fetal growth restriction and cardiovascular outcome in early human infancy: a prospective longitudinal study. Heart Vessels 31: 1504–1513, 2016. doi:10.1007/s00380-015-0742-5. 26386571

[48] Mikkola K, Leipälä J, Boldt T, Fellman V. Fetal growth restriction in preterm infants and cardiovascular function at five years of age. J Pediatr 151: 494–499 2007. doi:10.1016/j.jpeds.2007.04.030. 17961692

[49] Miyamoto K, Tsuboi T, Kokubu A, Suzumura H, Arisaka O. Assessment of contractility and myocardial function in small and appropriate for gestational age premature neonates using the stress-velocity relationship and tissue Doppler imaging immediately after birth. J Pediatr Endocrinol Metab 26: 999–1003, 2013. doi:10.1515/jpem-2012-0365. 23729605

[50] Montaldo P, Puzone S, Caredda E, Pugliese U, Inserra E, Cirillo G, Gicchino F, Campana G, Ursi D, Galdo F, Internicola M, Spagnuolo F, Carpentieri M, Capristo C, Marzuillo P, Del Giudice EM. Impact of intrauterine growth restriction on cerebral and renal oxygenation and perfusion during the first 3 days after birth. Sci Rep 12: 5067, 2022. doi:10.1038/s41598-022-09199-5. 35332251 PMC8948256

[51] Morsing E, Liuba P, Fellman V, Maršál K, Brodszki J. Cardiovascular function in children born very preterm after intrauterine growth restriction with severely abnormal umbilical artery blood flow. Eur J Prev Cardiol 21: 1257–1266, 2014. doi:10.1177/2047487313486044. 23613223

[52] Niewiadomska-Jarosik K, Zamojska J, Zamecznik A, Wosiak A, Jarosik P, Stańczyk J. Myocardial dysfunction in children with intrauterine growth restriction: an echocardiographic study. Cardiovasc J Afr 28: 36–39, 2017. doi:10.5830/CVJA-2016-053. 27925013 PMC5514352

[53] Olander RFW, Litwin L, Sundholm JKM, Sarkola T. Childhood cardiovascular morphology and function following abnormal fetal growth. Heart Vessels 37: 1618–1627, 2022. doi:10.1007/s00380-022-02064-5. 35426503 PMC9349157

[54] Olander RFW, Sundholm JKM, Ojala TH, Andersson S, Sarkola T. Differences in cardiac geometry in relation to body size among neonates with abnormal prenatal growth and body size at birth. Ultrasound Obstet Gynecol 56: 864–871, 2020. doi:10.1002/uog.21972. 31909531

[55] Patey O, Carvalho JS, Thilaganathan B. Perinatal changes in cardiac geometry and function in growth-restricted fetuses at term. Ultrasound Obstet Gynecol 53: 655–662, 2019. doi:10.1002/uog.19193. 30084123

[56] Rodriguez-Guerineau L, Perez-Cruz M, Gomez Roig MD, Cambra FJ, Carretero J, Prada F, Gómez O, Crispi F, Bartrons J. Cardiovascular adaptation to extrauterine life after intrauterine growth restriction. Cardiol Young 28: 284–291, 2018. doi:10.1017/S1047951117001949. 29081323

[57] Rodríguez-López M, Sepúlveda-Martínez Á, Bernardino G, Crovetto F, Pajuelo C, Sitges M, Bijnens B, Gratacós E, Crispi F. Cardiometabolic sex differences in adults born small for gestational age. Front Cardiovasc Med 10: 1223928, 2023. doi:10.3389/fcvm.2023.1223928. 37953765 PMC10634502

[58] Sarvari SI, Rodriguez-Lopez M, Nuñez-Garcia M, Sitges M, Sepulveda-Martinez A, Camara O, Butakoff C, Gratacos E, Bijnens B, Crispi F. Persistence of cardiac remodeling in preadolescents with fetal growth restriction. Circ Cardiovascular Imaging 10: e005270, 2017. doi:10.1161/CIRCIMAGING.116.005270. 28093413

[59] Sehgal A, Allison BJ, Gwini SM, Menahem S, Miller SL, Polglase GR. Vascular aging and cardiac maladaptation in growth-restricted preterm infants. J Perinatol 38: 92–97, 2018. doi:10.1038/jp.2017.135. 29120452

[60] Sehgal A, Allison BJ, Gwini SM, Miller SL, Polglase GR. Cardiac morphology and function in preterm growth restricted infants: relevance for clinical sequelae. J Pediatr 188: 128–134.e2, 2017. doi:10.1016/j.jpeds.2017.05.076. 28662946

[61] Sehgal A, Doctor T, Menahem S. Cardiac function and arterial biophysical properties in small for gestational age infants: postnatal manifestations of fetal programming. J Pediatr 163: 1296–1300, 2013. doi:10.1016/j.jpeds.2013.06.030. 23896189

[62] Sehgal A, Allison BJ, Miller SL, Polglase GR. Myocardial perfusion and function dichotomy in growth restricted preterm infants. J Dev Orig Health Dis 14: 302–310, 2023. doi:10.1017/S2040174422000630. 36408644

[63] Suciu LM, Giesinger RE, Mărginean C, Muntean M, Cucerea M, Făgărășan A, McNamara P. Comparative evaluation of echocardiography indices during the transition to extrauterine life between small and appropriate for gestational age infants. Front Pediatr 10: 1045242, 2022. doi:10.3389/fped.2022.1045242. 36727000 PMC9884809

[64] Vats K, Choudhary SK, Kumar D, Maria A, Bandopadhyay T. Myocardial performance index in term appropriate and small for gestational age neonates - a cross sectional study. J Neonatal Perinatal Med 14: 485–491, 2021. doi:10.3233/NPM-200621. 33523027

[65] Verma A, Suryawanshi P, Chetan C, Oka G, Singh Y, Kallimath A, Singh P, Garegrat R. A detailed echocardiographic evaluation of ventricular functions in stable full term small for gestational age babies. J Ultrasound 26: 117–127, 2023. doi:10.1007/s40477-022-00691-2. 35616853 PMC10063694

[66] Yiallourou SR, Wallace EM, Whatley C, Odoi A, Hollis S, Weichard AJ, Muthusamy JS, Varma S, Cameron J, Narayan O, Horne RSC. Sleep: a window into autonomic control in children born preterm and growth restricted. Sleep 40: zsx048, 2017. doi:10.1093/sleep/zsx048. 28419371

[67] Zaharie GC, Hasmasanu M, Blaga L, Matyas M, Muresan D, Bolboaca SD. Cardiac left heart morphology and function in newborns with intrauterine growth restriction: relevance for long-term assessment. Med Ultrason 21: 62–68, 2019. doi:10.11152/mu-1667. 30779833

[68] Alsaied T, Omar K, James JF, Hinton RB, Crombleholme TM, Habli M. Fetal origins of adult cardiac disease: a novel approach to prevent fetal growth restriction induced cardiac dysfunction using insulin like growth factor. Pediatr Res 81: 919–925, 2017. doi:10.1038/pr.2017.18. 28099426

[69] Coats LE, Bakrania BA, Bamrick-Fernandez DR, Ariatti AM, Rawls AZ, Ojeda NB, Alexander BT. Soluble guanylate cyclase stimulation in late gestation does not mitigate asymmetric intrauterine growth restriction or cardiovascular risk induced by placental ischemia in the rat. Am J Physiol Heart Circ Physiol 320: H1923–H1934, 2021. doi:10.1152/ajpheart.00033.2021. 33739156 PMC8428889

[70] Kumar P, Morton JS, Shah A, Do V, Sergi C, Serrano-Lomelin J, Davidge ST, Beker D, Levasseur J, Hornberger LK. Intrauterine exposure to chronic hypoxia in the rat leads to progressive diastolic function and increased aortic stiffness from early postnatal developmental stages. Physiol Rep 8–: e14327, 2020. doi:10.14814/phy2.14327. 31960611 PMC6971413

[71] Rueda-Clausen CF, Morton JS, Davidge ST. Effects of hypoxia-induced intrauterine growth restriction on cardiopulmonary structure and function during adulthood. Cardiovasc Res 81: 713–722, 2009. doi:10.1093/cvr/cvn341. 19088083

[72] Shah A, Matsumura N, Quon A, Morton JS, Dyck JRB, Davidge ST. Cardiovascular susceptibility to in vivo ischemic myocardial injury in male and female rat offspring exposed to prenatal hypoxia. Clin Sci (Lond) 131: 2303–2317, 2017. doi:10.1042/CS20171122. 28798077

[73] Thompson LP, Chen L, Polster BM, Pinkas G, Song H. Prenatal hypoxia impairs cardiac mitochondrial and ventricular function in guinea pig offspring in a sex-related manner. Am J Physiol Regul Integr Comp Physiol 315: R1232–R1241, 2018. doi:10.1152/ajpregu.00224.2018. 30365351 PMC6425638

[74] Schipper HS, de Ferranti S. Cardiovascular risk assessment and management for pediatricians. Pediatrics 150: e2022057957, 2022. doi:10.1542/peds.2022-057957. 36321395

[75] Rueda-Clausen CF, Morton JS, Dolinsky VW, Dyck JRB, Davidge ST. Synergistic effects of prenatal hypoxia and postnatal high-fat diet in the development of cardiovascular pathology in young rats. Am J Physiol Regul Integr Comp Physiol 303: R418–R426, 2012. doi:10.1152/ajpregu.00148.2012. 22739349

[76] Bencze M, Behuliak M, Zicha J. The impact of four different classes of anesthetics on the mechanisms of blood pressure regulation in normotensive and spontaneously hypertensive rats. Physiol Res 62: 471–478, 2013. doi:10.33549/physiolres.932637. 24020816

[77] Dai Y, Zhao D, Chen CK, Yap CH. Echocardiographic assessment of fetal cardiac function in the uterine artery ligation rat model of IUGR. Pediatr Res 90: 801–808, 2021. doi:10.1038/s41390-020-01356-8. 33504964 PMC8566221

[78] Gonzalez-Bulnes A, Astiz S, Parraguez VH, Garcia-Contreras C, Vazquez-Gomez M. Empowering translational research in fetal growth restriction: sheep and swine animal models. Curr Pharm Biotechnol 17: 848–855, 2016. doi:10.2174/1389201017666160519111529. 27194361

[79] Rizzo G, Mattioli C, Mappa I, Bitsadze V, Khizroeva J, Słodki M, Makatsarya A, D’Antonio F. Hemodynamic factors associated with fetal cardiac remodeling in late fetal growth restriction: a prospective study. J Perinat Med 47: 683–688, 2019. doi:10.1515/jpm-2019-0217. 31343984

[80] Shim CY. Arterial-cardiac interaction: the concept and implications. J Cardiovasc Ultrasound 19: 62–66, 2011. doi:10.4250/jcu.2011.19.2.62. 21860718 PMC3150697

[81] Clarke GD, Li J, Kuo AH, Moody AJ, Nathanielsz PW. Cardiac magnetic resonance imaging: insights into developmental programming and its consequences for aging. J Dev Orig Health Dis 12: 203–219, 2021. doi:10.1017/S2040174420001233. 33349289 PMC7987688

[82] Zhang F, Bryant KB, Moran AE, Zhang Y, Cohen JB, Bress AP, Sheppard JP, King JB, Derington CG, Weintraub WS, Kronish IM, Shea S, Bellows BK. Effectiveness of hypertension management strategies in SPRINT-Eligible US adults: a simulation study. J Am Heart Assoc 13: e032370, 2024. doi:10.1161/JAHA.123.032370. 38214272 PMC10926802

